# Early missed abortion is associated with villous angiogenesis via the HIF-1α/VEGF signaling pathway

**DOI:** 10.1007/s00404-018-4802-9

**Published:** 2018-06-27

**Authors:** Zhifu Zhi, Wenmei Yang, Liling Liu, XiaoLi Jiang, Lihong Pang

**Affiliations:** Department of Obstetrics and Gynecology, The First Affiliated Hospital of Guangxi Medical University, 6 Shuangyong Road, Nanning, Guangxi China

**Keywords:** HIF-1α, VEGF, Missed abortion, Angiogenesis

## Abstract

**Purpose:**

To analyze the effects of the hypoxia-inducible factor 1-alpha (HIF-1α)/vascular endothelial growth factor (VEGF) signaling pathway on villous angiogenesis in early missed abortion.

**Methods:**

Immunohistochemical assays were performed to detect the expression of micro-vessel density (MVD), HIF-1α, and VEGF in villous tissue samples from 30 missed abortions and 30 elective abortions in early pregnancy. We further analyzed the correlation between HIF-1α/VEGF and MVD. HTR8/SVneo cells were cultured under hypoxic (1%) or normoxic (20%) conditions, tube formation was investigated, and protein and mRNA level of HIF-1α/VEGF were determined using western blot and qRT-PCR. Finally, HIF-1α was knocked down with siRNA introduced into HTR8/SVneo cell line under hypoxia, and HIF-1α/VEGF expression and HTR8/SVneo tube formation were investigated.

**Results:**

The expression of HIF-1α, VEGF, and MVD was lower in the missed abortion than in the elective abortion group. Correlational analysis showed that the expression of HIF-1α and VEGF was positively correlated with MVD in both groups. The levels of HIF-1α/VEGF mRNA and protein in HTR8/SVneo cells were significantly enhanced under hypoxia. HIF-1α knockdown with siRNA inhibited HIF-1α/VEGF mRNA and protein levels of HTR8/SVneo cells induced by hypoxia. Tube formation of HTR8/SVneo cells was significantly enhanced in hypoxic culture and was inhibited by HIF-1α knockdown with siRNA.

**Conclusions:**

Our results reveal a novel role for HIF-1α/VEGF in regulating villous angiogenesis in early pregnancy and suggest that it may be a novel biomarker for missed abortion.

## Introduction

Angiogenesis is the most important factor involved in fetal and placental development. In the first trimester of pregnancy, differentiated placental trophoblast cells (termed extravillous cytotrophoblasts, EVT) invade maternal blood vessels, promote placental anchorage, and remodel the spiral arteries, resulting in increased blood flow toward the intravillous space [1, 2]. During placental development, some aspects of angiogenesis precede generation of the vasculature and lead to remodeling of the vascular plexus into a branched vascular tree so as to ensure increased nutritional and gas exchange [3]. Insufficient invasion by endovascular EVT predisposes the vasculature to perturbed remodeling, and impaired angiogenesis has been implicated in a number of pregnancy disorders that result in placental insufficiency, including miscarriages [4].

Placental angiogenesis is dependent upon various growth factors (including VEGF and its receptors) and on a number of signaling pathways [5, 6]. Maintenance of the attenuated oxygen milieu that normally presents in the first trimester placenta is essential for regulation of trophoblast function and crucial for early placental development. A low oxygen environment in early pregnancy may therefore be important in allowing EVT outgrowth expansion and promoting adequate placentation. HIF-1α is known to regulate cellular adaptation to hypoxic conditions [7]. Stabilized HIF-1α translocates to the nucleus and binds to the hypoxia-response elements of several target genes (such as VEGF), that are involved in the modulation of angiogenesis. VEGF has also been reported to be up-regulated under hypoxic conditions [8].

The HIF-1α/VEGF signaling pathway has been previously shown to play a crucial role in both angiogenesis and tumor growth [9, 10]. Although the role of HIF-1α/VEGF in villous angiogenesis is unknown, given the similarities between trophoblasts and cancer cells, we predict that its role in the placenta may be similar to its role in cancer, where it promotes angiogenesis. We herein hypothesized that HIF-1α/VEGF plays a critical role in villous angiogenesis.

To pursue this hypothesis, we evaluated the HIF-1α/VEGF expression and its correlation with MVD in villi from elective and missed abortions. We further examined the role of HIF-1α/VEGF in tube formation using the HTR8/SVneo cell line in vitro.

## Materials and methods

### Collection and processing of human villous tissues

Institutional review board approval was obtained from GXMU for the collection of villous tissue, and written informed consent was obtained from all patients. Villous tissue was obtained from women who requested elective induced abortions in early pregnancy (*n* = 30) and women who experienced missed abortions (*n* = 30), defined by an empty gestational sac or an embryo/fetus without cardiac activity after repeated transvaginal ultrasonographic scanning. The subjects who requested induced abortions showed a normal evolution in clinical and ultrasonographic assessments. All study subjects were at 6–10 weeks of gestation at the time of vacuum aspiration. Prior to inclusion in a study group, all patients underwent routine examinations to rule out any verifiable cause of missed abortion. None of the study participants had a history of pregnancy complications. Villous samples were collected when pregnancies were terminated by vacuum aspiration under mask anesthesia, and were immediately fixed in 10% neutral formalin for further immunohistochemical study.

### Culture of HTR8/SVneo cells

The HTR8/SVneo cell line was obtained from the Shanghai Cell Bank of the Chinese Academy of Sciences. Previous reports have shown that HTR-8/SVneo cells show endothelial cell-like behavior in their ability to form networks of tube-like structures when grown on matrigel [11, 12]. The cells were maintained in RPMI 1640 Medium (GIBCO, Invitrogen, NY, USA) supplemented with 5% fetal bovine serum (GIBCO) under normoxic or hypoxic conditions. Normoxic culture was performed in a standard incubator in an atmosphere containing 5% CO_2_ and 20% O_2_. Hypoxic-culture experiments were performed in a multigas incubator (ASTEC, Hukuoka,Japan) at 37 °C in an atmosphere balanced with 1% O_2_, 5% CO_2_ and 94% N_2_. After reaching ~ 80% confluency, the cells were subjected to hypoxic conditions in hypoxic chamber for 24 h. Cells cultured under hypoxic conditions were processed in the chamber itself to avoid any exposure to normoxic conditions. After incubation for 24 h, cells were collected for analysis within 1 min being removed from the chamber to prevent HIF1α protein degradation.

### Immunohistochemistry

Streptavidin-peroxidase (SP) immunohistochemical staining was adopted to detect the expression of HIF, VEGF, and MVD in villous tissue samples. In accordance with kit instructions (Zhongshan, Beijing, China), rabbit anti-human HIF, VEGF, and CD34 antibodies (dilution 1:200, ABclonal Biotechnology, USA) were used as primary antibodies. PBS buffer instead of the primary antibody was utilized as a negative control. Horseradish peroxidase-conjugated (HRP-conjugated) goat anti-rabbit IgG antibody (dilution1:800, Zhongshan, Beijing, China) was the secondary antibody. We then analyzed the samples using DAB substrate kit (Zhongshan, Beijing, China) according to the manufacturer’s instructions. All specimens were observed at five horizons and the average value was used for analysis. Photomicrographs were taken by light microscopy (Olympus BX-53, Tokyo, Japan) of five discontinuous visual fields under high magnification (400×) in each section in accordance with the conditions necessary for further software analysis. The images were analyzed using Image-Pro plus 6.0 software (Media Cybernetics, Rockville, MD) with respect to mean density (IOD/AIO). MVD was assessed by counting CD34-staining blood vessels at high power (400×) according to the method reported by Foote et al. [13]. Two pathologists determined the MVD score for the five most vascularized areas on the same section, and a mean count was obtained as the MVD.

### Quantitative reverse transcription-polymerase chain reaction (qRT-PCR)

We used real-time PCR to examine the mRNA expression levels of HIF-1α and VEGF in HTR8/SVneo cells. Total RNA was extracted from HTR8/SVneo cells with TRIzol reagent (Takara, Japan) according to the manufacturer’s protocol. The purity and concentration of RNA were assessed by ultramicrospectrophotometer (NanoDrop 2000, Thermos, USA). We performed reverse transcription in 20 μl of reaction system at 37 °C for 15 min and at 85 °C for 5 min. After reverse transcription of total RNA, the first-strand cDNA was then used as a template for the amplification of mRNA using quantitative real-time PCR with SYBR Green Master Mix (Roche, Germany). Human glyceraldehyde-3-phosphate dehydrogenase (GAPDH) was used as a control. QRT-PCR was performed using an ABI StepOne system (Applied BioSystems, USA) according to the manufacturer’s instructions, and the primers as follows: HIF-1α (forward: 5′-TTGCTCATCAGTTGCCACTTCC-3′, reverse: 5′-AGCAATTCATCTGTGCTTTCATGTC-3′), VEGF-A (forward: 5′-CGAGGGCCTGGAGTGTGT-3′, reverse: 5′-CGCATAATCTGCATGGTGATG-3′), GADPH (forward: 5′-GAGTCAACGGATTTGGTCGT-3′, reverse: 5′-GACAAGCTTCCCGTTCTCAG-3′). The PCR reaction was performed in 20 μl of reaction system as follows: 95 °C for 30 s, and 40 cycles of 95 °C for 5 s and 60 °C for 30 s. The expression of a specific gene was determined with the 2^−ΔΔct^ method.

### Western blot

Cells were washed twice with cold PBS and lysed in ice-cold lysis buffer with added protease inhibitor cocktail (Roche, Germany). The protein concentration was then determined with a BCA kit (Thermo Scientific, USA). A volume of 20 μg of each sample was separated on SDS-PAGE, and then transferred to PVDF membranes, and blocked with 5% nonfat dry milk for 1 h. Membranes were incubated overnight at 4 °C to primary antibodies anti-HIF-1α, anti-VEGF, or anti-GADPH at 1:200 dilutions (ABclonal Biotechnology, USA). The membranes were then washed twice for 10 min each in 0.1% Tween phosphate buffered saline solution (PBST), and probed with goat anti-rabbit IR-Dye 670 or 800 cw-labeled secondary antisera (CST, USA) in 0.1% Tween, and 0.01% SDS LiCor blocking buffer for 1 h at room temperature. Washes were repeated after secondary labeling, washing twice for 10 min in PBST, and then the membranes were placed in water. Membranes were imaged using a LiCor Odyssey scanner. Boxes were manually placed around each band of interest, which returned near-infrared fluorescent values of raw intensity, with intra-lane background subtracted using Odyssey 3.0 analytical software (LiCor, Lincoln, NE).

### SiRNA transfection

The siRNA transfection was performed using Lipofectamine RNAiMAX transfection reagent (Roche, Germany) according to the manufacturer’s instructions. One day before transfection, 25,000 cells were plated in 250 μl of growth medium without antibiotics per well in a 24-well plate. The cell density reached 30–50% confluency at the time of transfection. Cells were transfected with either siRNA duplexes that targeted HIF-1α or with a negative control siRNA. The cells were incubated for 24 h at 37 °C under hypoxic conditions until the time of the assay for gene knockdown. Knockdown was evaluated using qRT-PCR of HIF-1α, and lead to a > 90% decrease in HIF-1α mRNA expression in HTR8/SVneo cell lines.

### Tube formation

Growth factor-reduced matrigel (BD Biosciences, USA) was diluted with serum-free medium at a ratio of 1:2 with cooled pipettes and distributed in a 24-well plate (150 ml per well) on ice. After the matrigel mixture solidified, HTR8/SVneo cells (1.0 × 10^5^ per well) with various pretreatments were gently added to each of the triplicate wells followed by normaxic or hypoxic culture for 12 h. Photomicrographs of three random fields of view were taken using an inverted phase-contrast microscope (Olympus, Tokyo, Japan). To evaluate the angiogenic process, we quantified the number of branching points. The number of branching points that generated at least three tubules was then counted in independent experiments each performed in duplicate.

### Statistical analysis

For statistical analysis, SPSS for Windows version 18.0 (SPSS, Chicago, IL, USA) was used. All data are presented as mean ± SD. Comparisons between groups were analyzed by Student’s *t* test or one-way ANOVA followed by Tukey’s test for multiple comparisons. For correlational analyses, the Pearson correlation analysis was used. *P* value < 0.05 was considered to be statistically significant. All experiments in vitro were performed in triplicate.

## Results

### HIF-1α/VEGF is correlated with villous angiogenesis

**Fig. 1:**
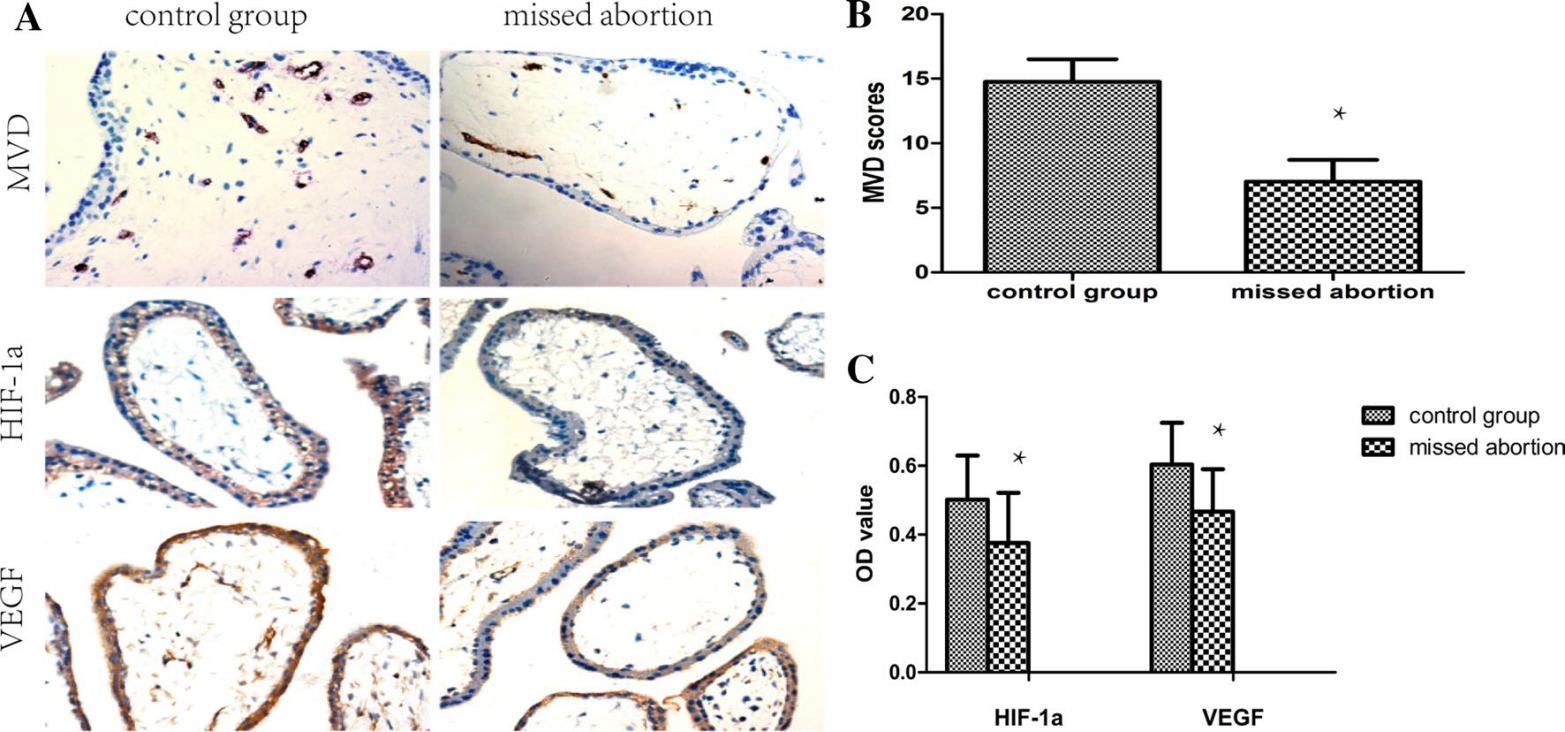
Immunohistochemical staining for MVD, HIF-1α, and VEGF observed in the cytoplasm of syncytiotrophoblast cells and cytotrophoblast cells (**a**). (×400) Tissues from missed abortions showed lower MVD than with normal pregnancy (**b**). Normal pregnancy showed a high expression of HIF-1α and VEGF (**c**). *P < 0.05 vs. control group

**Fig. 2:**
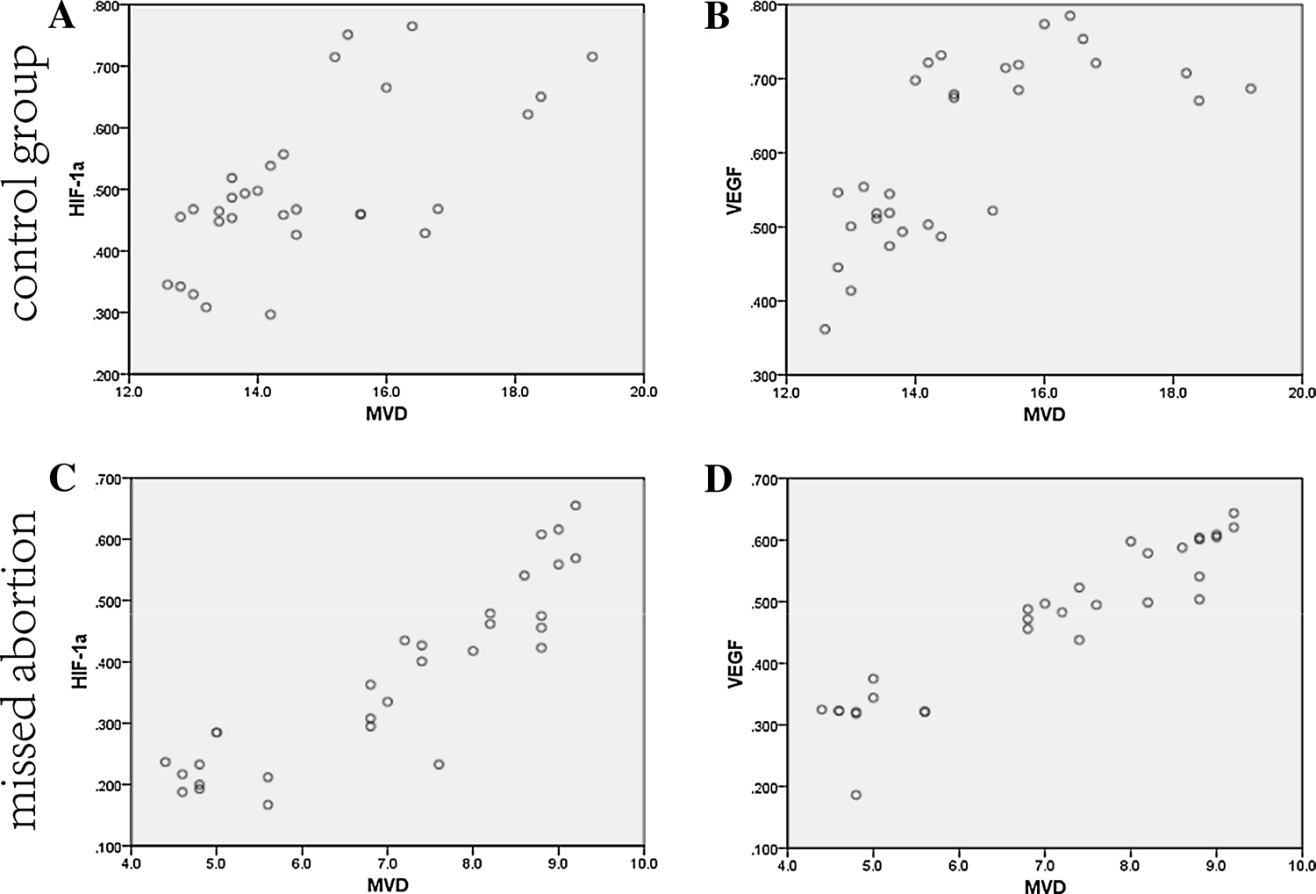
MVD was correlated positively with HIF-1α and VEGF in the normal pregnancy group (**a**, **b**). MVD was also correlated positively with HIF-1α and VEGF in the missed abortion group (**c**, **d**)

There were no significant differences in age, gestational age, gravidity, parity, and induced abortion rates between the two groups. As shown in Fig. 1, the expression of HIF-1α and VEGF in missed abortion tissues was significantly lower than in tissues from normal pregnancies. In addition, there were lower levels of MVD with missed abortion than in elective abortion. HIF-1α and VEGF expression were significantly correlated with MVD in both groups (shown in Fig. 2). Therefore, we showed that a positive correlation exists between HIF-1α/VEGF and villous angiogenesis. The HIF-1α/VEGF signaling pathway stimulated angiogenesis in trophoblast cells.

### Hypoxia induces mRNA and protein expression of HIF-1α and VEGF

**Fig. 3:**
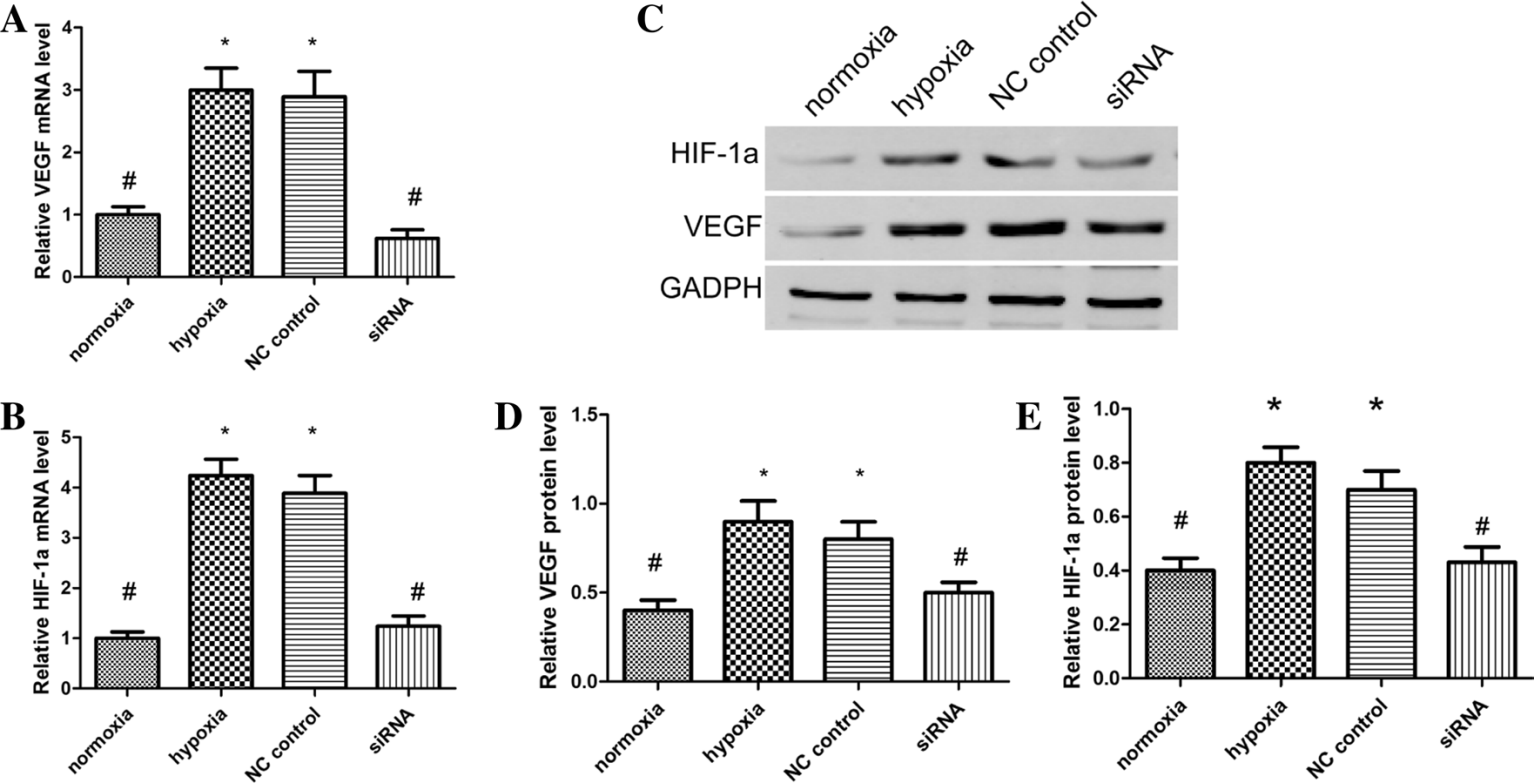
Changes in HIF-1α and VEGF mRNA levels in each group as measured by quantitative PCR (**a**, **b**). Protein expression of HIF-1α and VEGF in differentially treated HTR8/SVneo cells as measured by western blot analyst (**c**). Protein expression relative to GAPDH of HIF-1α ratio (**d**), and VEGF ratio (**e**); Values are means mean ± standard deviation (*n* = 3 for each group). Negative control siRNA as NC control. *P < 0.05 vs. normoxic group; ^#^*P* < 0.05 vs hypoxic group

To determine the expression of HIF-1α/VEGF in the HTR8/SVneo cell line, we measured mRNA and protein levels for HIF-1α and VEGF with qRT-PCR and WB. The HIF-1α and VEGF mRNA levels were significantly increased in hypoxia compared to normoxia (Fig. 3). In addition, hypoxic treatment significantly increased HIF-1α and VEGF protein levels compared to normoxic conditions. When we further examined HIF-1α effects on VEGF, we observed that HTR8/SVneo transfection with HIF-1α siRNA reduced both HIF-1α and VEGF mRNA and protein levels with hypoxia.

### HIF-1α and VEGF promote HTR8/SVneo tube formation

**Fig. 4:**
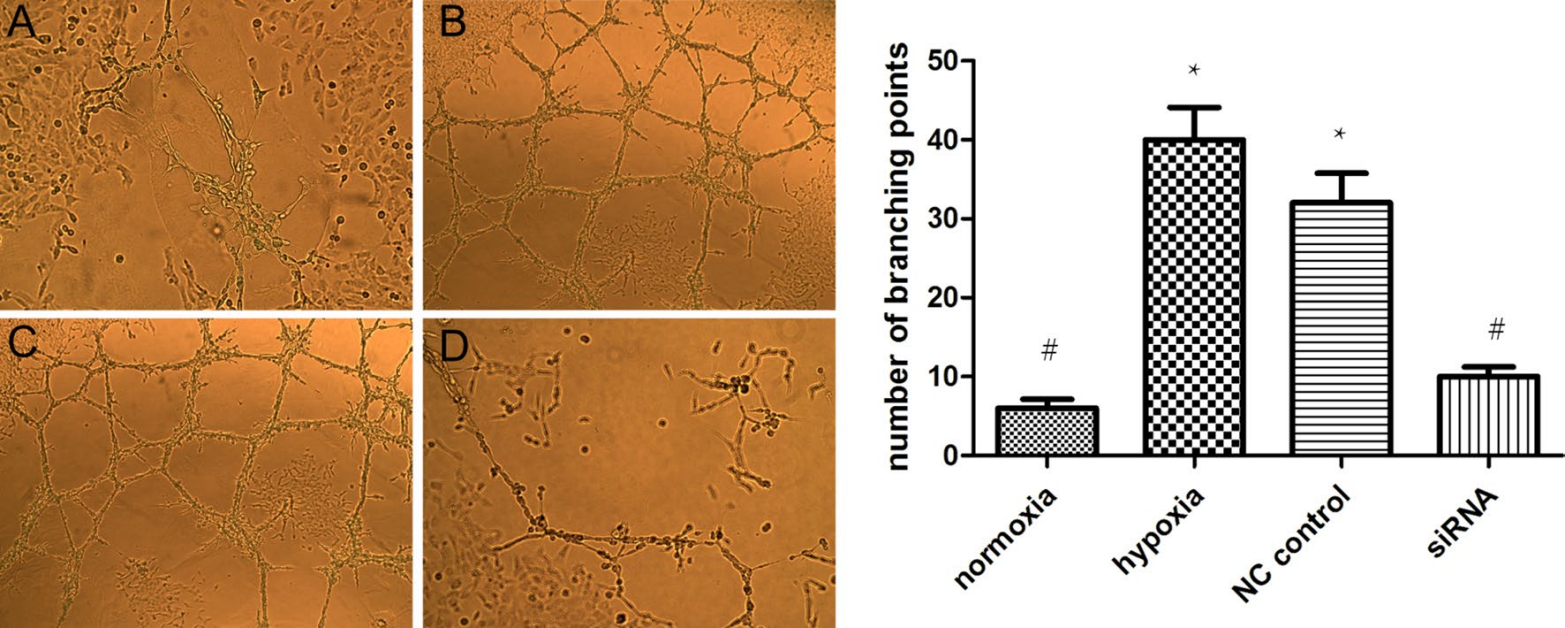
Tube formation in HTR-8/SVneo cells. Representative photomicrographs after cultured for 12 h were shown, ×100. (**a** Normoxia, **b** hypoxia, **c** negative control siRNA as NC control, **d** siRNA). The columns indicate the number of branching points

We found that tube formation in HTR8/SVneo cells was higher under hypoxic relative to normoxic conditions. The results of the in vitro tube formation experiment showed that microtubule density of the siRNA group was significantly lower than that of the negative control group (*P* < 0.01, Fig. 4). The results for this section confirmed that the ability for vasculogenic mimicry by cell lines was inhibited after HIF-1α silencing.

## Discussion

In this study, we showed a novel role for HIF-1 a/VEGF in placental development. We demonstrated that HIF-1 a/VEGF regulated villous angiogenesis, and loss of HIF within human villous trophoblasts correlated with missed abortion. These results suggest that HIF-1 a/VEGF functions to regulate placentation in humans, and that dysregulation of HIF-1 a/VEGF may contribute to the development of missed abortion.

Remodeling of maternal spiral arteries involves trophoblast invasion and EVT differentiation, whereby EVT forms the linings of the uterine vessels [2]. Spiral arterioles are blocked by invading endovascular trophoblasts that restrict blood flow and thereby sustain a low oxygen environment of 1–2% oxygen. In humans, first trimester chorionic villi show detectable HIF-1α levels [7]. The human placenta affords a unique environment in terms of oxygenation, as it undergoes a transition from a hypoxic to a more oxygenated environment, and this physiologic switch in oxygen tension and dynamic HIF-α signaling is required for placental development. Placental vascular development can be affected via activation of the HIF-1α signaling pathway and its targets (such as VEGF), leading to altered placental vascularization, the VEGF family is known to regulate placental angiogenesis and maternal spiral artery remodeling. Within trophoblast cells, HIF-1α/VEGF appears to regulate numerous functions, including vasculogenesis, and plays a role in regulating the hypoxic response. Collectively, the results of the present study define an important regulatory relationship between angiogenesis at the villi and the HIF-1α/VEGF signaling pathway.

It has been demonstrated that HIF-1 protein levels can be regulated at three different steps: transcription, translation, and protein stability. Under hypoxic conditions, HIF-1α expression increases due to diminished ubiquitination and degradation. Our in vitro studies suggest that hypoxia modulates total HIF-1α/VEGF mRNA and protein level. Our findings also indicate that HIF-1α acts upstream of VEGF, and that lack of its expression leads to placental hypoxia because of a failure of appropriate placental angiogenesis. Nüsken et al. reported an increase in both HIF-1α protein and mRNA under hypoxia in primary human trophoblasts isolated from normal term placentae [14]. Wang et al. also achieved the same results in the hypoxia-treated human trophoblast cell line HTR8/SVneo [15]. In contrast, Rajakumar and Conrad demonstrated that in early human placenta, regulation of these transcription factors by hypoxia occurs at the level of protein and not mRNA [16]. The reasons for these apparently conflicting results on HIF-1α mRNA regulation under hypoxia are not clear, but may be related to different exposure times and cell types used in the respective studies.

Missed abortion is a common and distressing complication of early pregnancy, and is defined as a pregnancy that no longer develops in which the cervical os remains closed [17]. Many etiologies contribute to missed abortion, including genetic factors, endocrine factors, and anatomical factors. However, since the pathophysiologic mechanisms underlying 50% missed abortion cases are not fully understood. More recent attention has focused on abnormal angiogenesis as an important cause of missed abortion [18, 19]. In recent years, progress in the field of insufficient angiogenesis has offered new insights into the possible causes of this condition, and has revealed novel avenues for research into its prevention and treatment.

Angiogenesis is a critical step in placental and fetal development, and it is hypothesized that vascular deficiencies may lead to early miscarriage. In fact, it is now well established that VEGF is the most potent factor involved in angiogenesis, with several studies demonstrating that attenuated expression of VEGF is related to early pregnancy loss [20–22]. During the first trimester of human pregnancy, HIF-1α is known to play an essential role in trophoblasts physiology under hypoxic conditions [23]. An intriguing aspect to the present work is the suggestion that HIF-1α/VEGF may play an important role in the development of placental insufficiency-related pregnancy disorders, such as missed abortion. These findings may be somewhat limited by a small sample size and by the lack of information on appropriate animal models for experiments in vivo.

The altered placental vascularization seen in pregnancy-related disorders, such as missed abortion is incompletely understood, therefore, determining the mechanisms by which placental vascularization occurs will be important in identifying markers, and for targeting therapies in missed abortion. Although we predict that altered HIF-1 a/VEGF may play a role in the development of placental insufficiency, additional experiments will be necessary to determine its penetrance.
